# Impact of Physical Rehabilitation on Bone Biomarkers in Non-Metastatic Breast Cancer Women: A Systematic Review and Meta-Analysis

**DOI:** 10.3390/ijms24020921

**Published:** 2023-01-04

**Authors:** Alessandro de Sire, Lorenzo Lippi, Nicola Marotta, Arianna Folli, Dario Calafiore, Stefano Moalli, Alessio Turco, Antonio Ammendolia, Nicola Fusco, Marco Invernizzi

**Affiliations:** 1 Physical and Rehabilitative Medicine, Department of Medical and Surgical Sciences, University of Catanzaro “Magna Graecia”, Viale Europa, 88100 Catanzaro, Italy; 2 Physical and Rehabilitative Medicine, Department of Health Sciences, University of Eastern Piedmont “A. Avogadro”, 28100 Novara, Italy; 3 Dipartimento Attività Integrate Ricerca e Innovazione (DAIRI), Translational Medicine, Azienda Ospedaliera SS. Antonio e Biagio e Cesare Arrigo, 15121 Alessandria, Italy; 4 Physical Medicine and Rehabilitation Unit, Department of Neurosciences, ASST Carlo Poma, 46100 Mantova, Italy; 5 Division of Pathology, IEO, European Institute of Oncology, IRCCS, Via Giuseppe Ripamonti 435, 20141 Milan, Italy; 6 Department of Oncology and Hemato-Oncology, University of Milan, Via Festa del Perdono 7, 20122 Milan, Italy

**Keywords:** osteoporosis, biomarkers, rehabilitation, physical exercise, precision medicine, breast cancer

## Abstract

Rehabilitation might improve bone health in breast cancer (BC) patients, but the effects on bone biomarkers are still debated. Thus, this meta-analysis of randomized controlled trials (RCTs) aims at characterizing the impact of rehabilitation on bone health biomarkers in BC survivors. On 2 May 2022, PubMed, Scopus, Web of Science, Cochrane, and PEDro were systematically searched for RCTs assessing bone biomarker modifications induced by physical exercise in BC survivors. The quality assessment was performed with the Jadad scale and the Cochrane risk-of-bias tool for randomized trials (RoBv.2). Trial registration number: CRD42022329766. Ten studies were included for a total of 873 patients. The meta-analysis showed overall significant mean difference percentage decrease in collagen type 1 cross-linked N-telopeptide (NTX) serum level [ES: −11.65 (−21.13, −2.17), *p* = 0.02)] and an increase in bone-specific alkaline phosphatase (BSAP) levels [ES: +6.09 (1.56, 10.62). According to the Jadad scale, eight RCTs were considered high-quality studies. Four studies showed a low overall risk of bias, according to RoBv.2. The significant effects of rehabilitation on bone biomarkers suggested a possible implication for a precision medicine approach targeting bone remodeling. Future research might clarify the role of bone biomarkers monitoring in rehabilitation management of cancer treatment induced bone-loss.

## 1. Introduction

Breast cancer (BC) is the most common malignancy in women, with an increasing incidence worldwide [1]. In the last years, the mortality rate related to BC significantly decreased due to the advances in screening programs, early diagnosis, and therapeutical interventions [2]. However, in response to the progressive increase in BC survivors, the prevalence of long terms disabling consequences in these women is steadily increasing, along with the growing need for therapeutic intervention addressing physical and psychosocial impairment that characterizes the so-called “survivorship issues” in BC women [3,4,5].

In this scenario, cancer treatment-induced bone loss (CTIBL) is a common consequence of cancer treatments affecting several BC survivors [6,7,8]. Hormonal therapy (HT) is the gold standard adjuvant therapy for postmenopausal women with hormone receptor (HR)-positive non-metastatic BC [6,7,8]. However, HT negatively affects bone mineral density (BMD) due to residual serum endogenous estrogen levels deprivation, leading to a significant increase in fragility fracture risk [8,9,10,11]. Concurrently, chemotherapy has been related to an unspecific increase in bone resorption, while corticosteroids drug administration has been widely documented to have detrimental consequences on bone health due to a reduction in both bone formation and osteoblast and osteocyte viability [12,13]. Therefore, several pharmacological approaches have been proposed to counter CTIBL, with growing evidence emphasizing the need for precise risk stratification to better guide clinicians in anti-resorptive drug prescription to preserve bone health and reduce the risk of fragility fractures [14,15,16]. On the other hand, lifestyle medicine plays a pivotal role in the multicomponent management of bone and muscle health status in non-metastatic BC survivors, with several relevant guidelines recommending the implementation of a comprehensive CTIBL management, including a calcium-enriched diet, oral supplementation of 1000–2000 IU of vitamin D3 daily, and physical exercise to counteract a potential osteosarcopenia [17,18,19].

More in-detail, physical exercise might prevent bone loss, increase BMD, and reduce fall risk due to the well-known improvement in physical function, balance control, and muscle strength [20]. In this context, hip and trunk muscles are considered as main targets for physical training aiming at stimulating exercise-induced osteogenic effects [21]. To date, several studies supported the role of rehabilitation and physical exercise in improving bone health and quality of life in post-menopausal osteoporotic women [22,23]. More in-detail, the recent systematic review and meta-analysis performed by Kemmler et al. [22] underlined that different exercise modalities might positively affect BMD at the lumbar spine, femoral neck, or total hip site in postmenopausal women [22]. However, to date, the role of rehabilitation in preventing and managing CTIBL is far from being fully understood, whereas recent research is now focusing on the implementation of a precision medicine approach to rehabilitation interventions in accordance with the recent trend of biomarker-based treatment of cancer patients [24,25]. Thus, despite the mechanisms underpinning CTIBL being far from understood in detail, targeting specific molecular modifications might be considered a promising therapeutical approach in the precision medicine management of bone health in BC survivors. On the other hand, evidence supporting precise monitoring of biological effects of rehabilitation interventions is still lacking, not only in cancer patients, but also in other fields of medicine. Moreover, to the best of our knowledge, no previous systematic reviews assessed the effects of different exercise modalities on bone biomarkers in BC survivors.

Therefore, the aim of this systematic review and meta-analysis was to assess the impact of physical rehabilitation interventions on bone biomarker modifications in non-metastatic BC patients. This might potentially guide physicians and future research to more precise monitoring of bone health and CTIBL treatment in these women.

## 2. Methods

### 2.1. Registration

This systematic review of randomized controlled trials (RCTs) was performed according to the Preferred Reporting Items for Systematic Reviews and Meta-analyses (PRISMA) statement [26]. Preliminary research on the international prospective register of systematic reviews (PROSPERO) was performed to evaluate if other similar works were in progress. No similar review was identified, thus the study was submitted to PROSPERO and accepted on 2 May 2022 (available at https://www.crd.york.ac.uk/prospero, accessed on 16 December 2022, registration number CRD42022329766).

### 2.2. Search Strategy

Five databases on medical sciences and physical and rehabilitation medicine were systematically searched on 10 May 2022. Two investigators independently searched PubMed/Medline, Scopus, Cochrane Central Register of Controlled Trials (CENTRAL), Physiotherapy Evidence Database (PEDro), and Web of Science (WOS). Duplicates were excluded independently by two investigators. Further details of the search strategy are reported in Table 1. 

### 2.3. Selection Criteria

Review question was characterized by the following PICO model [27]:

- (P) Participants: adult women (18 years and older) with non-metastatic BC.
- Intervention: any rehabilitation treatment administered before, during, or after chemotherapy and/or radiotherapy treatments.
- (C) Comparator: any comparator including pharmacological, non-pharmacological, or no treatment.
- (O) Outcome: primary outcomes were bone metabolic biomarkers. Secondary outcomes were other bone health outcomes, including bone mineral density or trabecular bone score.

RCTs were considered eligible if published in international peer-reviewed journals. The exclusion criteria were the following: (i) language other than English; (ii) studies involving animals; (iii) pregnancy; (iv) clinical instability; and (v) conference abstracts, masters, or doctorate theses.

### 2.4. Study Screening and Eligibility Assessment

After duplication removal, two investigators independently reviewed the title and abstracts of the retrieved records to choose relevant articles. Discordances between the two authors were solved by collegial discussion. A third reviewer was asked if consensus was not possible. All the reports that met the inclusion and exclusion criteria were screened in full text by the same investigators, and the records that met the eligibility criteria were included in the data extraction. Any disagreements between the two investigators were discussed with a third reviewer to reach consensus.

### 2.5. Data Extraction and Synthesis

All data were assessed and extracted independently from full-text documents into Word by two authors. Any disagreement between the two reviewers was solved by collegial discussion among the Authors. In case of disagreement, a third author was asked. All the data extracted were summarized in tables.

Data synthesis was performed for the following data: (1) authors; (2) journal; (3) publication year; (4) nationality; (5) participants characteristics [number, mean age and age range, Body Mass Index (BMI)]; (6) tumor characteristics; (7) treatment characteristics; (8) interventions’ characteristics (type of rehabilitative treatment, number of sessions, intensity, duration of intervention); (9) comparator; (10) outcomes; and (11) main findings. 

### 2.6. Meta-Analysis

The meta-analysis was performed by Revman 5.4.0 (The Cochrane Collaboration, 2020, Boston, MA, USA). Changes in serum markers were displayed as mean difference percentage (MD%) and standard deviation (SD). The heterogeneity among comparisons was estimated by the Chi-squared and I^2^ statistic tests. An I^2^ > 75% determined significant heterogeneity across the articles. In the event of considerable heterogeneity, a random-effects model was adopted to determine the pooled estimates with the effect size (ES) and 95% confidence interval (CI). Missing means and SDs were estimated from medians, ranges, and interquartile ranges (IQRs) using the method introduced by Hozo et al. [28].

### 2.7. Quality Assessment and Risk of Bias

The quality of the studies included was assessed independently by two authors, according to the Jadad scale [29]. Discordances were solved by discussion between the authors or by asking a third reviewer. The items assessed were the following (i) random sequence generation; (ii) appropriate randomization; (iii) blinding of participants or personnel; (iv) blinding of outcome assessors; and (v) withdrawals and dropouts. A Jadad score between 3 to 5 points was considered high quality.

The Cochrane risk-of-bias tool for randomized trials (RoBv.2) [30] was implemented for risk of bias assessment. The following domains were assessed by RoBv.2: (i) randomization process; (ii) deviations from the intended interventions; (iii) missing outcome data; (iv) measurements of the outcome; and (v) selection of the reported results. According to these items, bias was classified as low, high, or having some concerns.

## 3. Results

**Figure 1:**
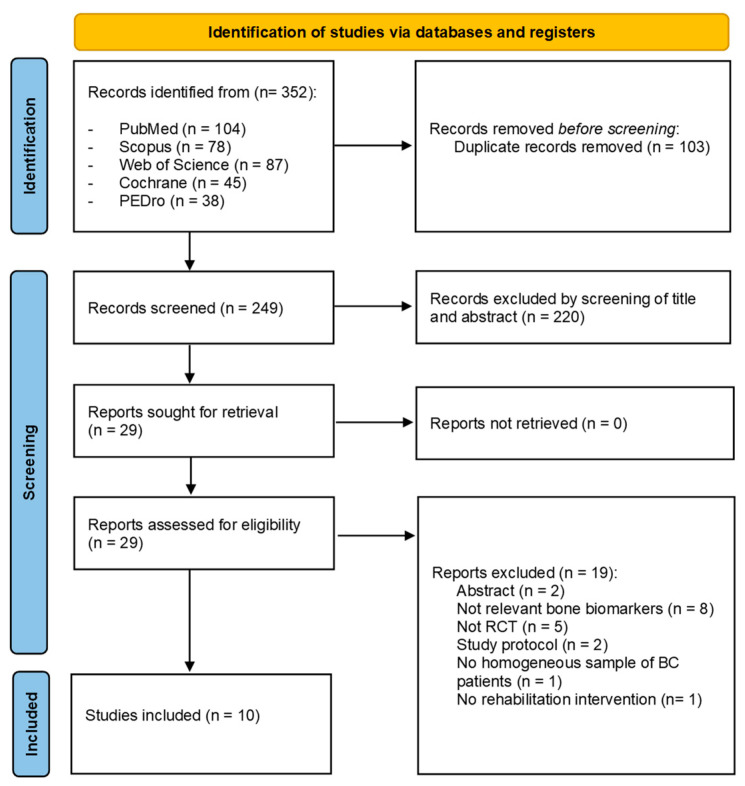
PRISMA 2020 flow diagram.

Through our search strategy, 352 records were identified from the five databases. After duplication removal, 249 studies were assessed for eligibility and screened for title and abstract. Therefore, 220 records were excluded, and 29 full-text records were assessed for eligibility. Nineteen records were excluded for inconsistency with the eligibility criteria (two were only abstracts, eight studies did not assess relevant bone biomarkers, five studies were not RCT, two studies were RCT protocols, one study did not assess a homogeneous sample of BC patients, and one study did not assess rehabilitation intervention). The studies assessed in full text and the reasons for exclusions are presented in detail in Supplementary Table S1. Lastly, 10 studies were included in the present work [31,32,33,34,35,36,37,38,39,40]. Figure 1 shows the PRISMA 2020 flow diagram of the search process in detail.

### 3.1. Study Characteristics

The RCTs included were published between 2010 [36,38] and 2018 [31,32,33,35]. The nationalities of the studies included in this review were as follows: seven studies (70%) were conducted in the USA [33,35,36,37,38,39,40], one (10%) was conducted in Australia [31], one (10%) was conducted in Brazil [32], and one (10%) was conducted in South Korea [34]. All the characteristics of the included studies are shown in detail in Table 2.

### 3.2. Participants

In the present review, 873 subjects (100% females) were assessed in the included studies. More in-detail, 442 BC patients were included in the intervention groups, while 431 BC patients were included in the control groups. The ages of the subjects included ranged from 45.2 ± 5.9 years [37] to 66.6 ± 9.6 years [32]. The body composition was assessed by BMI, and it ranged from 23.3 ± 4.3 kg/m^2^ [34] to 33.5 ± 5.5 kg/m^2^ [33]. However, it should be noted that one study [38] reported the number of patients per range of age (≤60, >60 years) and BMI (≤25, >25 kg/m^2^).

The cancer stages ranged from 0 [34,35,36,39] to IIIB [36], but it should be noted that one study [31] did not characterize the cancer stage, although including only non-metastatic BC patients. 

Breast cancer surgery was characterized by three studies as mastectomy and breast conservative surgery. None of the studies included characterized axillary surgery [31,32,33,34,35,36,37,38,39,40]. More in-detail, mastectomy prevalence ranged between 52.2% [3] and 57% [5] in interventional groups, while in control groups it ranged between 20% [4] and 41.2% [2]. 

Radiation therapy administration ranged between 60.9% [34] and 100% [35] in intervention groups and between 61.1% [40] and 100% [35] in control groups. However, 4fourstudies did not report radiation therapy administration [32,36,37,38]. 

Chemotherapy administration ranged between 54.5% [35] and 100% [37] in intervention groups, while it ranged between 59.3% [39] and 100% [37] in control groups. Five studies did not characterize chemotherapy administration [31,33,36,38,40]. 

Hormonal therapy was administered to 100% of study participants in three studies [31,32,35]. Among the other studies, hormone therapy administrations in the intervention groups ranged between 42% [36] and 78.3% [34], while in the control group it ranged between 53.7% [39] and 85% [34]. On the other hand, two studies did not characterize endocrine therapy administration [33,38].

Table 2 shows further details on cancer stage and cancer treatments received in each study included.

### 3.3. Control Groups

Control groups included BC patients that underwent usual care, vitamin supplementation, pharmacological treatment, stretching and relaxation exercises, and/or psychosocial support therapy. More in-detail, rehabilitation treatment was compared to usual care in three studies [31,33,35], standard treatment combined with psychosocial support in one study [36], stretching and relaxation techniques in three studies [32,39,40], monthly health newsletter in one study [37], and pharmacological intervention with risedronate, calcium, and vitamin D administration in one study [38].

The groups have been characterized in detail in Table 3.

### 3.4. Rehabilitation Therapy Interventions

In the present review, the rehabilitation therapy intervention included resistance exercise training (RET), combined exercise training (CET—aerobic exercise training combined with RET), RET combined with impact exercise training (IET), Thai Chi Chuan, and whole-body vibration (WBV) training. More in-detail, five studies [32,33,34,35,37] assessed CET protocols, making CET the most studied training modality. Only one study focused on the effects of RET [38], while two studies assessed RET combined with IET [39,40]. The remaining two studies assessed the effects of WBV training [31] and Thai Chi Chuan exercise programs [36]. Interestingly, Peppone et al.’s (2018) study assigned the BC patients to three different control groups, assessing separately the efficacy of CET in addition to oral vitamin supplementation, only CET, and only oral vitamin supplementation. Five of the training protocols were supervised by operators [31,32,33,36,37], while three studies assessed training protocols performed with initial supervision followed by home-based sessions [38,39,40]. Lastly, 2 studies assessed home-based protocols [34,35]. All the studies assessed exercise protocols in BC patients after chemotherapy and/or radiotherapy [31,32,33,34,35,36,37,38,39,40].

### 3.5. Primary Outcome—Bone Biomarkers Modifications

In the present review were included RCTs assessing the biological effect of different rehabilitation programs in terms of modification of concentration of the markers described below.

- Collagen type 1 cross-linked N-telopeptide (NTX) was assessed in four studies [33,35,36,38], but significant changes were reported only in one study [38]. In particular, Waltman et al. [38] reported significant changes (*p* < 0.05) in both the intervention group (RET + risedronate, calcium, and Vitamin D) and the control group (risedronate, calcium, and vitamin D).
- Urinary NTX excretion normalized to creatinine (NTX/Cr) was assessed in three studies [31,34,37]. However, only Tabatabai et al. [37] reported significant changes (*p* < 0.05) in both intervention (CET) and control groups (monthly health newsletter).
- Procollagen type I N-terminal propeptide (P1NP) was assessed in two studies [31,37], reporting significant changes in one study [37]. More in-detail, Tabatabai et al. [37] reported a significant decrease (*p* < 0.05) in both intervention and control groups.
- Collagen Type I C-Telopeptide (CTX) was assessed in three studies [32,33,37]. Interestingly, Tabatabai et al. [41] reported a significant decrease in both groups (*p* < 0.05). However, no significant between-group differences were reported in the studies considered [32,33,37].
- Osteocalcin was assessed in five papers [32,33,37,39,40]; out of these, two studies [32,33] reported a significant increase (both *p* < 0.05) in the intervention group after CET, while Winters-Stone et al., 2011, [39] reported a significative inter-group difference after RET combined with IET (*p* = 0.01). Lastly, Tabatabai et al. [37] reported a significant decrease in both intervention (CET) and control groups.
- Bone-specific alkaline phosphatase (BSAP) was assessed in four papers [33,35,36,38]; among these studies, Dieli-Conwright et al. [33] reported a significant increase in serum concentration in the CET intervention group compared to intervention; concurrently, Waltman et al. [38] reported a reduction in both the intervention group (RET + risedronate, calcium, and Vitamin D) and control group (risedronate, calcium, and Vitamin D). The remaining studies did not report a significant modification of BSAP values (*p* > 0.05).
- Deoxypyridinoline change in serum level was assessed in two studies [39,40], although neither reported a significative intra- or intergroup difference (*p* > 0.05).
- Receptor activator of nuclear factor (RANK) was assessed by Dieli-Conwright et al. [33], but the study did not report significant changes (*p* > 0.05).
- Receptor activator of nuclear factor ligand (RANKL) was assessed by Dieli-Conwright et al. [33], without reporting significant changes (*p* > 0.05).

**Figure 2:**
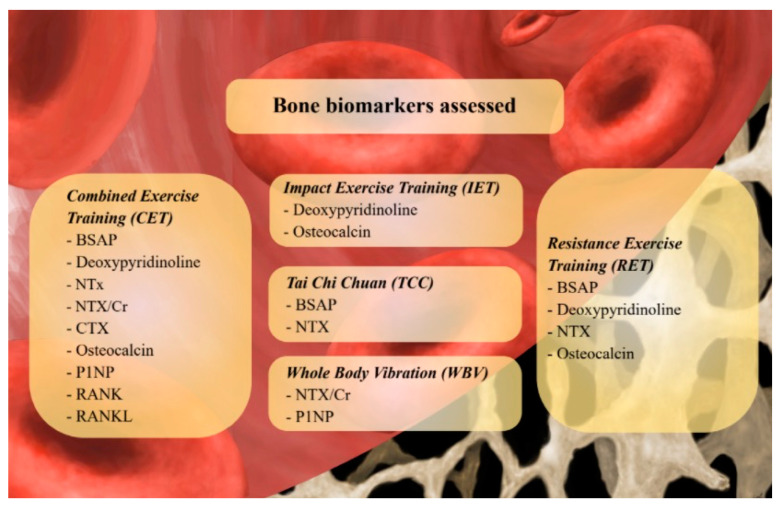
Bone biomarkers proposed in the current literature to assess the effects of different exercise modalities. *Abbreviations*: BSAP: Bone Specific Alcaline Phosphatase; CTX: Cross-linked Collagen Type I C-telopeptide; NTX: Crosslinked Collagen Type I N-telopeptide; NTX/Cr: Cross-linked Collagen Type I N-telopeptide/creatinine ratio; RANK(L): Receptor Activator of Nuclear Factor KB (Ligand); P1NP: Procollagen Type 1 intact N-terminal.

Figure 2 graphically summarized the bone biomarkers proposed in the current literature to assess the effects of different exercise modalities.

### 3.6. Secondary Outcomes—Bone Mineral Density

Most of the papers [32,33,34,37,38,39,40] included in our study assessed BMD; further details are reported below:

- Whole body BMD was assessed in two studies [32,33,37], without reporting significant changes in intergroup analysis.
- Lumbar spine BMD was assessed in seven studies [32,33,34,37,38,39,40]; more in-detail, Winters-Stone et al., 2011, [39] underlined significant changes (*p* < 0.01) in the intergroup analysis after RET combined with IET intervention after 12 months. Similarly, Waltman et al. [38] reported a percentage mean difference significant both in the intervention group (RET + risedronate, calcium, and vitamin D) and the control group (risedronate, calcium, and vitamin D) (both *p* < 0.05). Interestingly, Tabatabai et al. [37] reported a significant mean decrease in the control group, which received only a monthly health newsletter (*p* = 0.03).
- Total Hip BMD was assessed in seven studies [32,33,34,37,38,39,40]. Waltman et al. [38] reported significant changes in both intervention group (RET + risedronate, calcium, and Vitamin D) and control group (risedronate, calcium, and Vitamin D).
- Trochanter BMD was assessed in four studies [32,33,39,40], but no significant changes were reported.
- Femoral neck BMD was assessed in six studies [33,34,37,38,39,40], without showing significant changes.
- Radius (33% length) BMD was assessed by Waltman et al. [38], without reporting significant changes.
- Total radius BMD was assessed by Waltman et al. [38], with no significant changes after the intervention.

Table 4 summarized the primary and secondary outcomes of the present review.

### 3.7. Meta-Analysis

**Figure 3:**
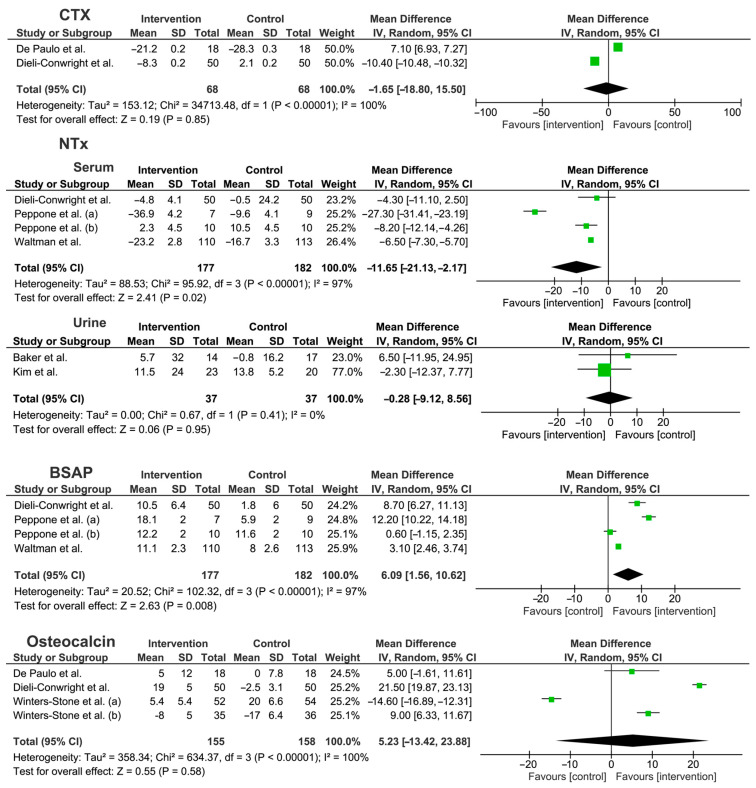
Meta-analysis of the studies included [31,32,33,34,35,36,38,39,40]. Abbreviations BSAP: bone specific alkaline phosphatase; CI: confidence interval; CTX: C-telopeptides of type I collagen; NTX: crosslinked N-telopeptides of type I collagen; SD: standard deviation.

A meta-analysis was performed to underline the effects of different exercise interventions on bone metabolism biomarkers of non-metastatic BC patients, showing an overall significant MD% decrease in NTX serum level [ES: −11.65 (−21.13, −2.17), *p* = 0.02)] and an increase in BSAP levels [ES: +6.09 (1.56, 10.62), *p* = 0.008)]. On the other hand, no significant differences were found for urinary NTX, CTX, and osteocalcin markers. Percentage differences between intervention and control group provided by Waltman et al. [38] were used to adapt the data related to the combined intervention (exercise combined with risedronate versus risedronate alone). A random-effects model was adopted since the low number of RCTs included and the high heterogeneity of rehabilitation intervention (for further details see Figure 3).

The study by Tabatabai et al. [37] was excluded from the meta-analysis because numerical data were not reported.

### 3.8. Quality Assessment and Risk of Bias

According to the Jadad scale, eight (80%) RCTs were considered high-quality studies [31,33,34,35,36,37,39,40]. Lower quality was found in two (30%) studies [32,38] due to missing information about randomization methods or blindness of data assessors. On the other hand, it should be noted that blindness of participants and personnel was not achievable in all the studies included due to the intrinsic nature of the rehabilitative treatment. Table 5 showed in detail the score of each subitem of the Jadad scale for the RCTs included.

**Figure 4:**
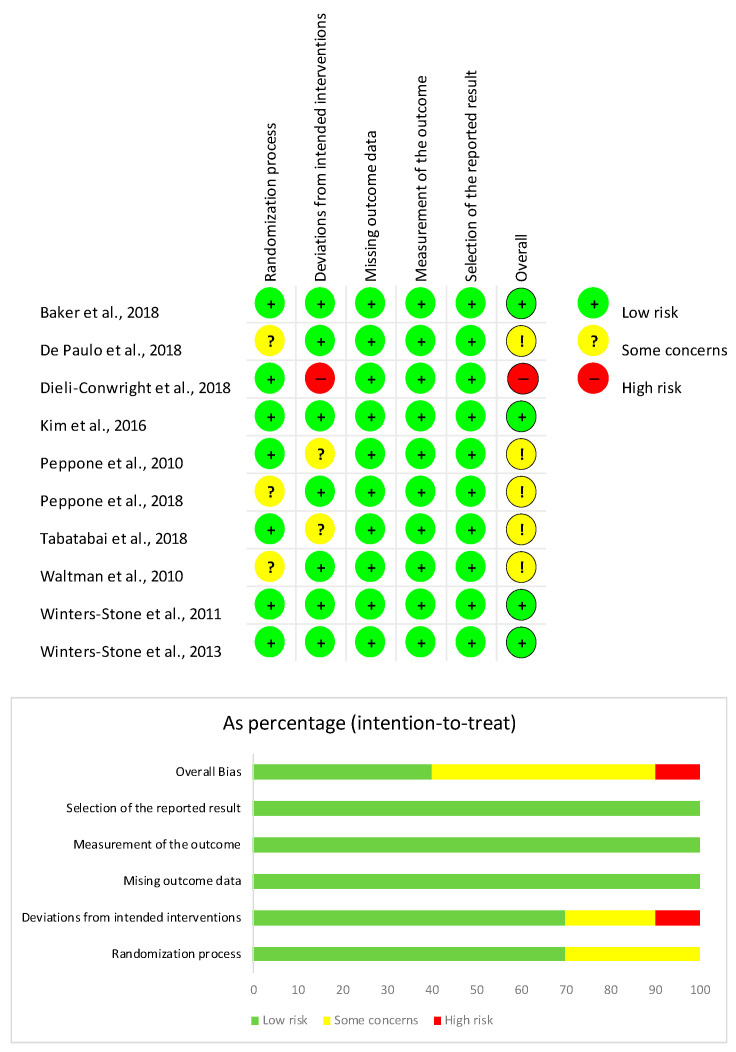
Risk of bias of the studies included [31,32,33,34,35,36,37,38,39,40] according to the RoB2.

The risk of bias was assessed by RoBv.2, reporting 4 studies [31,34,39,40] (40%) with a low overall risk of bias. Two studies (20%) [33] showed some concerns in the second domain for deviations from intended interventions due to the missing appropriate analysis for effect of assignment to intervention. These concerns lead to an overall medium risk of bias. Lastly, 1 study [33] (10%) showed a high risk of bias for exclusion of five women from analysis after the intervention, resulting in a high overall risk of bias (see Figure 4).

## 4. Discussion

In recent years, the long-term management of BC survivors has gained a rising interest in both clinical and research settings, considering the growing prevalence of cancer disabling sequelae affecting these women. Several papers highlight the need for structured and tailored rehabilitation intervention to improve both physical and psychosocial well-being of BC women [41,42]. In this scenario, CTIBL is widespread disabling condition in cancer patients and physical exercise plays a pivotal role in its prevention due to the multifaceted effects on the whole musculoskeletal system, improving both BMD and reducing the risk of falling in patients at high risk of fragility fracture [43,44,45]. However, to date, several questions are still open about the precise biological effects of physical exercise on bone metabolism and health since the complex multilevel interactions characterizing CTIBL in non-metastatic BC survivors. In light of these considerations, this meta-analysis of RCTs assessed the effects of different exercise modalities on currently available bone biomarkers, providing a broad overview about the evidence supporting biomarker implementation in the clinical setting in order to guide physicians in a precise prescription of individualized rehabilitation plans.

Interestingly, our meta-analysis showed significant effects in terms of NTX serum level [ES: −11.65 (−21.13, −2.17), *p* = 0.02)]. However, it should be noted that the results of individual studies were not significant in the majority of the RCT included. This limitation might be partly related to the small sample of the studies considered and the low effect size, given that the results of the pooled sample showed significant differences in terms of NTX. NTX is one of the most important biomarkers to assess bone resorption [46,47,48]. Its levels in bloodstream reflect the liberation of peptides produced by degradation of osteoid (composed mostly of collagen); in this context, its serum levels might quantify the rate of bone resorption [49], also considering the role that might play in repairing bone and nerves [50,51]. Moreover, the recent systematic review by Migliorini et al. [52] found a significant association between NTX serum level and lower spine and hip BMD, suggesting that a NTX serum level might reflect an increased bone turnover, leading to a reduction in both BMD and T-score. Interestingly, the qualitative synthesis identified one study [38] reporting significant changes in NTX serum levels after RET intervention, suggesting that RET might be the most promising modality in inducing NTX serum level modifications. However, it should be noted that urinary NTX excretion did not show significant changes in the meta-analysis [37].

In recent years, the International Osteoporosis Foundation (IOF) and the International Federation of Clinical Chemistry and Laboratory Medicine (IFCC) identified NTX and CTX as the most promising bone biomarkers in the clinical setting of bone pathological conditions, including osteoporosis [53,54]. Despite positive results being reported in NTX serum level modifications, the results of our meta-analysis did not show significant differences in terms of CTX modifications after physical exercise programs. However, few studies [37,38] assessed CTX serum levels in BC women undergoing physical exercise programs. Therefore, these data might be significantly affected by the low number of studies currently available in the literature.

Considering bone deposition biomarkers, BSAP is a bone marker of bone formation that showed a significant increase after exercise therapy interventions in BC patients (BSAP levels [ES: +6.09 (1.56, 10.62) *p* = 0.008)]. Approximately 50% of BSAP is produced from the skeletal system in subjects with normal liver function. However, no studies assessing BSAP reported the liver function status of study participants. Thus, to date, CET or RET seems to be the exercise modality most supported in improving blood levels of BSAP in BC survivors [33,38].

Similarly, osteocalcin is selectively secreted by osteoblast and is considered a bone marker to assess bone anabolic activity [55]. In particular, γ-carboxylate osteocalcin has great affinity for hydroxyapatite and is commonly stored in bone tissues [56]. Osteocalcin decarboxylation promotes its endocrine activity as bone-derived hormone, with recent studies highlighting its role in glucose metabolism [55,56,57]. Our results highlighted a significant improvement in terms of osteocalcin serum level in two studies [32,33] assessing CET or RET combined with IET. On the other hand, the meta-analysis did not show significant benefits of exercise in terms of osteocalcin.

In the last two decades, increasing interest has been raised in both RANK and RANK-L pathways, two crucial pharmacological targets in the management of osteoporosis. More in-detail, RANK is a transmembrane receptor involved in the signaling pathway regulating osteoclast differentiation and activation. To date, this pathway is the main target of the monoclonal antibody Denosumab, exerting its antiresorptive action by blocking the interaction between RANK and RANK-L, with consequent inhibition of osteoclast activity [16,58]. On the other hand, RANK is not monitored in the current clinical practice, and there is a lack of studies in terms of modifications after physical exercise training in BC survivors [33].

In addition, RANK monitoring might be crucially affected by the pharmacological therapies commonly administered to prevent CTIBL [59,60,61]. Therefore, RANK and RANK-L should not be considered bona fide biomarkers to assess the biological effects of physical exercise in BC patients. Lastly, no evidence supports their integration in a precision medicine approach focusing on bone health management in BC survivors.

Taken together, our findings showed positive results of certain specific bone biomarkers reflecting the effects of physical exercise on bone health in BC survivors. However, conflicting data were reported about BMD modifications induced by physical exercise in these patients. More in-detail, three studies showed positive results in terms of lumbar spine BMD improvement after physical exercise interventions [37,38,39]. These findings might be probably related to the trabecular structure of vertebra that is metabolically more active and might be more sensible to mechanical stimuli promoting bone formation at the lumbar spine level [62,63]. In addition, RET alone or combined with IET might be the most promising therapeutic approach to improve lumbar spine BMD [37,38,39]. Unfortunately, few studies included in the present work assess T-score or Z-score, probably due to the short-term follow-up period and the non-pharmacological intervention that might provide little changes related to the short terms follow-up and instrumental errors, highlighting another gap of knowledge in the current. In this scenario, previous studies suggested that a multimodal approach, including different exercise modalities, might be the most suitable option to improve bone health in patients with osteoporosis [64,65,66]. Moreover, the recent systematic review by Marini et al. [65] suggested RET and IET as the most promising exercise modalities to reduce the risk of fracture.

On the other hand, several controversies are still open about the macroscopical effects of physical exercise on BMD, and previous systematic reviews and meta-analyses reported insufficient evidence to support a superior effect of one specific exercise modality [67,68]. However, it should be noted that the currently available literature focused on standardized exercise programs without focusing on the biological effects of physical exercise in an individualized rehabilitation plan. Moreover, our systematic review did not identify studies considering a precision medicine approach based on bone remodeling biomarkers to tailor physical exercise programs to the patient’s characteristics.

Taken together, our findings underlined that bone biomarkers might be significantly affected by physical exercise and could be possibly implemented in monitoring tailored rehabilitation interventions aimed at treating CTIBL in BC survivors. To the best of our knowledge, this is the first systematic review focusing on the effects of different exercise modalities on bone biomarkers in non-metastatic BC survivors. In the era of precision medicine, a biomarker-based approach might have a role in improving the comprehensive rehabilitation management of these women, including not only physical exercise, but also antiresorptive drugs in patients at high risk of fracture to maximize outcomes and reduce the disability and socio-sanitary costs of fragility fractures [69,70,71]. In addition, due to the widely documented effects of physical exercise on oxidative stress and inflammation, a precise multitarget rehabilitation intervention might not only improve bone health, but also have potential interaction with malignant transformation and tumor progression pathways in BC patients [72,73,74,75,76].

Besides these considerations, we are aware that this study is not free from limitations. More in-detail, the low number of studies included, and the small sample size might limit the strengths of our conclusions. On the other hand, our results reflect the papers currently available about this topic in five different databases and put to light a gap of knowledge in the current literature. However, it should be noted that the sample size assessed allows us to obtain significant results in quantitative synthesis. On the other hand, the heterogeneity of the study population, exercise characteristics, and bone biomarkers might represent the main limitations of the present review. To reduce potential bias related to this issue, we provided a detailed qualitative synthesis to characterize the heterogeneity of the studies. Moreover, meta-analysis has been performed in subgroup analysis for bone biomarkers, limiting the potential implications of their heterogeneity. Lastly, only the study by Waltman et al. [38] assessed the effects of physical exercise in a comprehensive rehabilitation approach to CTIBL, including also pharmacological treatments. In this context, it should be noted that antiresorptive drugs should be integrated into the bone health management of BC survivors receiving AIs in accordance with the most recent guidelines [10,17,18,77]. Given the antiresorptive drugs’ effects on bone metabolisms, further good quality studies are needed to better characterize the impact of physical exercise on bone biomarkers in BC patients treated with antiresorptive drugs for preventing CTIBL.

However, our findings might be a catalyst for a deeper understanding of biological processes regulating the multilevel interaction between physical exercise, bone remodeling, and CTIBL. Future research should focus on the precise characterization of physical exercise programs, highlighting the biological differences induced by a comprehensive rehabilitation plan.

## 5. Conclusions

Physical exercise is one of the main non-pharmacological interventions counteracting CTIBL in non-metastatic BC survivors. However, to date, no previous systematic review assessed the effects of physical exercise on circulating bone biomarkers, and the effects of different exercise modalities on bone biomarkers are still debated.

Taken together, the results of the meta-analysis suggested significant effects of rehabilitation in terms of NTX and BSAP levels modifications, even though the heterogeneity of the study results might limit the strength of our conclusions. However, our data might have potential implications for the prescription of physical exercise targeting bone remodeling in patients with non-metastatic BC. Future research might clarify the role of bone biomarker monitoring in the comprehensive management of CTIBL to optimize the synergistic role of non-pharmacological and pharmacological approaches in promoting bone health in BC survivors.

## Figures and Tables

**Table 1:** Search strategy.

PubMed:	
((“breast neoplasms” [MeSH Terms] OR (“breast” [All Fields] AND “neoplasms” [All Fields]) OR “breast neoplasms” [All Fields] OR (“breast” [All Fields] AND “cancer” [All Fields]) OR “breast cancer” [All Fields] OR “breast tumor” [MeSH Terms] OR (“breast” [All Fields] AND “tumor” [All Fields]) OR “breast tumor” [All Fields])) AND ((“rehabilitant” [All Fields] OR “rehabilitants” [All Fields] OR “rehabilitate” [All Fields] OR “rehabilitated” [All Fields] OR “rehabilitates” [All Fields] OR “rehabilitating” [All Fields] OR “rehabilitation” [MeSH Terms] OR “rehabilitation” [All Fields] OR “rehabilitations” [All Fields] OR “rehabilitative” [All Fields] OR “rehabilitation” [MeSH Subheading] OR “rehabilitation s” [All Fields] OR “rehabilitational” [All Fields] OR “rehabilitator” [All Fields] OR “rehabilitators” [All Fields] OR “exercise” [MeSH Terms] OR “exercise” [All Fields] OR (“physical” [All Fields] AND “exercise” [All Fields]) OR “physical exercise” [All Fields] OR “training” [All Fields] OR “train” [All Fields] OR “train s” [All Fields] OR “trained” [All Fields] OR “training s” [All Fields] OR “trainings” [All Fields] OR “trains” [All Fields])) AND ((“bone and bones” [MeSH Terms] OR (“bone” [All Fields] AND “bones” [All Fields]) OR “bone and bones” [All Fields] OR “bone” [All Fields]) AND (“biomarker s” [All Fields] OR “biomarkers” [MeSH Terms] OR “biomarkers” [All Fields] OR “biomarker” [All Fields]) OR (“bone remodeling” [MeSH Terms] OR (“bone” [All Fields] AND “remodeling” [All Fields]) OR “bone remodeling” [All Fields] OR (“bone” [All Fields] AND “turnover” [All Fields]) OR “bone turnover” [All Fields] OR “osteogenesis” [MeSH Terms] OR “osteogenesis” [All Fields] OR (“bone” [All Fields] AND “formation” [All Fields]) OR “bone formation” [All Fields] OR “bone resorption” [MeSH Terms] OR (“bone” [All Fields] AND “resorption” [All Fields]) OR “bone resorption” [All Fields]))	
**Scopus:**	
TITLE-ABS-KEY (((((((breast AND neoplasms) OR (breast AND cancer) OR (breast AND tumor)) AND ((rehabilitation) OR (rehabilitation AND therapy) OR (exercise) OR (physical AND exercise)) AND ((bone AND (biomarker OR biomarkers)) OR (bone AND remodelling) OR (bone AND turnover)))))))	
**Web of Science:**	
(((((breast neoplasms) OR (breast cancer) OR (breast tumor)) AND ((rehabilitation) OR (rehabilitation therapy) OR (exercise) OR (physical exercise)) AND ((bone biomarker) OR (bone biomarkers) OR (bone remodelling) OR (bone turnover)))))	
**Cochrane:**	
ID	Search
#1	MeSH descriptor: [breast neoplasms] explode all trees
#2	MeSH descriptor: [breast cancer] explode all trees
#3	MeSH descriptor: [breast tumor] explode all trees
#4	MeSH descriptor: [rehabilitation] explode all trees
#5	MeSH descriptor: [rehabilitation therapy] explode all trees
#6	MeSH descriptor: [exercise] explode all trees
#7	MeSH descriptor: [physical exercise] explode all trees
#8	MeSH descriptor: [bone biomarker] explode all trees
#9	MeSH descriptor: [bone biomarkers] explode all trees
#10	MeSH descriptor: [bone remodelling] explode all trees
#11	MeSH descriptor: [bone turnover] explode all trees
#12	(#1 OR #2 OR #3) AND (#4 OR #5 OR #6 OR #7) AND (#8 OR #10 OR #11)
**PEDro:**	
breast cancer, exercise, bone *	

**Table 2:** Main characteristics of the study population.

Authors Journal Year	Participants				Cancer Treatments			
	Sample Size	Age (Years)	BMI (kg/m^2^)	Cancer Characteristics	Breast Surgery	Chemotherapy	Radiation Therapy	Hormonal Therapy
Baker et al. *Integr. Cancer Ther.* 2018 [31]	N = 31 IG: 14 CG: 17	N = 61.6 ± 8.3 IG: 61.6 ± 9.2 CG: 61.6 ± 7.8	N = 28.8 ± 4.7 IG: 28.4 ± 3.9 CG: 29.1 ± 5.3	Non-metastatic breast cancer	NR	NR	NR	100%
De Paulo et al. *Exp. Gerontol.* 2018 [32]	N = 36 IG: 18 CG: 18	N = NR IG: 63.2 ± 7.1 CG: 66.6 ± 9.6	N = NR IG: 28.9 ± 5.2 CG: 31.5 ± 6.3	*Stage I* IG: 50% CG: 58.8% *Stage II* IG: 33.3% CG: 23.6% *Stage IIIA* IG: 17.6% CG: 17.6%	*Partial Mastectomy* IG: 47.4% CG: 58.8% *Total Mastectomy* IG: 52.6% CG: 41.2%	NR	NR	100%
Dieli-Conwright et al. *Breast Cancer Res.* 2018 [33]	N = 100 IG: 50 CG: 50	N = 53.5 ± 10.4 IG: NR CG: NR	N = 33.5 ± 5.5 IG: NR CG: NR	*Stage I*: 40% *Stage II*: 38%	NR	Chemotherapy and radiotherapy (76%)	Chemotherapy and radiotherapy (76%)	*NR*
Kim et al. *Cancer Nurs.* 2016 [34]	N = 43 IG: 23 CG: 20	N = NR IG: 55.7 ± 5.3 CG: 56.3 ± 6.7	N = NR IG: 23.3 ± 4.3 CG: 23.4 ± 2.5	*Stage 0-I*: IG: 7 (31.8%) CG: 12 (60.0) *Stage II-III*: IG: 15 (68.2) CG: 8 (40.0)	*Mastectomy*: IG: 12 (52.2%) CG: 14 (20.0%) *Breast-conserving* *surgery*: IG: 11 (47.8%) CG: 16 (80.0%)	IG: 18 (78.3%) CG: 15 (75.0%)	IG: 14 (60.9%) CG: 16 (80.0%)	*Selective estrogen receptor modulator* IG: 13 (56.6%) CG: 9 (45%) *Aromatase inhibitor* IG: 5 (21.7%) CG: 8 (40.0%)
Peppone et al. *Clin Breast Cancer* 2010 [36]	N = 16 IG: 7 CG: 9	N = NR IG: 53.8 CG: 52.6	N = NR IG: 25.8 CG: 24.2	*Breast Cancer* *Stage 0-IIIB*	*Mastectomy* IG: 57% CG: 33.3% *Lumpectomy* IG: 43% CG: 66.7%	NR	NR	IG: 42.9% CG: 66.7%
Peppone et al. *Support. Care Cancer* 2018 [35]	N = 41 IG1 (exercise + supplementation): 10 IG2 (exercise): 10 IG3 (supplementation): 10 CG: 11	N = 53.5 ± 7.8 IG1: ≤48 = 18.2% 49–57 = 54.5% ≥58 = 27.3% IG2: ≤48 = 10.0% 49–57 = 40.0% ≥58 = 50.0% IG3: ≤48 = 50.0% 49–57 = 20.0% ≥58 = 30.0% CG: ≤48 = 60.0% 49–57 = 20.0% ≥58 = 20.0%	N = NR IG1: 31.3 IG2: 32.4 IG3: 28.1 CG: 28.4 *p* = 0.29	*Stage 0-I* IG1: 63.6% IG2: 20% IG3: 50% CG: 30% *Stage II* IG1: 27.3% IG2: 60% IG3: 40% CG: 40% *Stage III* IG1: 9.1% IG2: 20% IG3: 10% CG: 30%	NR	IG1: 54.5% IG2: 70% IG3: 70% CG: 70%	IG1: 100% IG2: 100% IG3: 100% CG: 100%	*Tamoxifen* IG1: 36.4% IG2: 44.4% IG3: 60% CG: 70% *Aromatase inhibitor* IG1: 63.6% IG2: 55.6% IG3: 40% CG: 30%
Tabatabai et al. *J. Clin. Endocrinol. Metab.* 2016 [37]	N = 206 IG: 103 CG: 103	N = NR IG: 46.0 ± 5.7 CG: 45.2 ± 5.9	N = NR IG: 26.2 ± 6.0 CG: 25.7 ± 6.7	*Non-metastatic Breast Cancer*	NR	100%	*NR*	*Tamoxifen* IG: *49%* CG: *72%*
Waltman et al. *Osteoporos Int.* 2010 [38]	N = 223 IG: 110 CG: 113	*≤60 years* IG: 55 CG: 61 *>60 years* IG: 45 CG: 39	*≤25* IG: 42 CG: 34 *>25* IG: 58 CG: 66	*Stage I–II breast cancer*	NR	NR	NR	NR
Winters-Stone et al. *Breast Cancer Res Treat.* 2011 [39]	N = 106 IG: 52 CG: 54	N = NR IG: 62.3 ± 6.7 CG: 62.2 ± 6.7	N = NR IG: 29.5 ± 5.8 CG: 29.5 ± 5.6	Stage 0–IIIA breast cancer *Stage 0* IG: 7.7% CG: 3.7% *Stage I* IG: 38.5% CG: 40.7% *Stage II* IG: 48.1% CG: 35.2% *Stage IIIa* IG: 1.9% CG: 9.3%	NR	IG: 61.5% CG: 59.3%	IG: 92.3% CG: 83.3%	IG: 59.6% CG: 53.7%
Winters-Stone et al. *Osteoporos Int.* 2013 [40]	N = 71 IG: 35 CG: 36	N = NR IG: 46.5 ± 5 CG: 46.4 ± 4.9	N = NR IG: 27 ± 5.4 CG: 25.8 ± 4.6	*Stage I* IG: 22.9% CG: 33.3% *Stage II* IG: 65.7% CG: 50% *Stage III* IG: 11.4% CG: 16.7%	NR	NR	IG: 62.9% CG: 61.1%	*Aromatase inhibitors* IG: 40% CG: 30.6% *SERM* IG: 37.1% CG: 44.4%

Abbreviations: CG: Control Group; IG: Intervention Group; NR: not reported.

**Table 3:** Main characteristics of the study interventions.

Authors Journal Year	Intervention								Comparator
	*Type of Activity*	*Exercise Modality*	*Protocol Duration*	*Frequency*	*Volume (Session)*	*Intensity*	*Supervision or Home-Based*	*Timing*	
Baker et al. *Integr. Cancer Ther.* 2018 [31]	WBV	WBV	12 weeks	Three sessions/wk	20 min	Low-frequency, low-magnitude vibration (30 Hz, 0.1 mm, 0.3 g)	Supervised	After chemotherapy and/or radiotherapy	Usual care
De Paulo et al. *Exp. Gerontol.* 2018 [32]	CET	– RET on weight machines: seated cable row, bench press, leg extension, leg press, and leg curl/bridge, abdominal, and plank exercises), – AET: treadmill	36 weeks	Three sessions/wk	CET: 100 min (5 min warm-up, 55 min of RET, 30 min of AET, 10 min cooldown)	NR	Supervised	After chemotherapy and/or radiotherapy	*Type of activity*: low intensity stretching and relaxation *Exercise modality*: stretching and relaxation exercises *Protocol duration*: 36 weeks *Frequency*: Two sessions/wk *Volume (session)*: 45 min per session *Intensity*: low intensity *Supervised* *After chemotherapy and/or radiotherapy*
Dieli-Conwright et al. *Breast Cancer Res.* 2018 [33]	CET	– RET in circuit with no rest periods (leg press and chest press, lunges and seated row, leg extensions and tricep extensions, and leg flexion and bicep curls); – AET: self-selected, i.e., treadmill walking/running, rowing machine	16 weeks	Three sessions/wk	5 min warm-up; Days one and three: approximately 80 min of CET; Day two: AET progressively increased from 30 min up to 50 min; 5-min cool down at 40–50% estimated VO_2_ max	80% of estimated 1-RM for lower body exercises and 60% estimated 1-RM for upper body, keeping target HR in AET at 60–80% of maximum, increased every 4 weeks	Supervised	After chemotherapy and/or radiotherapy	Usual care
Kim et al. *Cancer Nurs.* 2016 [34]	CET	– AET: walking – RET: Thera-Bands including five upper-body and four lower-body exercises targeting the major muscle groups	6 months	AET: three nonconsecutive days; RET: two to three sessions/wk	AET: minimum 150 min/wk; RET: Two sets of 8–10 repetition	AET: 11 to 13 perceived exertions on a six to 20 point scale; RT: low to moderate intensity	Home-based + Telephone counseling	After chemotherapy and/or radiotherapy + Supplementation with 500 mg calcium and 1000 IU vitamin D	Supplementation with 500 mg calcium and 1000 IU vitamin D
Peppone et al. *Clin Breast Cancer* 2010 [36]	thai chi chuan (TCC) exercises	Warm up, stretches, Chi Kung; TCC sessions consisting of a 15-move short form sequence of Yang-style of TCC; cool down with regulatory breathing, imagery, and meditation	12 weeks	Three sessions/wk	NR	10 min warm up; 40 min TCC sessions; 10 min cool down	Supervised	After chemotherapy and/or radiotherapy	Usual care + Behavioral coping strategies, cohort support and group unity. Patients instructed not to change their pattern of physical activity in any manner for the duration of the intervention
Peppone et al. *Support. Care Cancer* 2018 [35]	CET	– RET: resistance bands exercises: squats, side bends, leg extensions, leg curls, chest press, rows, toe raises, overhead press, biceps curls, triceps extensions – AET: walking	12 weeks	RET: Three times per week with at least one rest day between the sessions; AET: Seven days/wk	RET: starting with an individually determined number of sets (7–10 repetitions) up to three sets for each exercise; AET: walking with a pedometer increasing daily the step count by 5–10%, up to 12,000 steps	Moderate intensity (60–70% HR)	Home-based	After chemotherapy and/or radiotherapy + Calcitriol supplementation	Usual care
Tabatabai et al. *J. Clin. Endocrinol. Metab.* 2016 [37]	CET	– RET: exercises for hamstrings, quadriceps, gluteus, thigh abductors, thigh adductors, pectoralis, latissimus dorsi, biceps, triceps, deltoids, erector spinae and rhomboids. – AET: self-selected exercises with cardiovascular machines, walking, running, or bicycling	12 months	Three times per week	AET: 20–30 min; RET: one set of 13 exercises, completing eight repetitions, up to two sets of exercises with 8–12 repetitions	NR	Supervised	After chemotherapy and/or radiotherapy	Monthly health newsletter
Waltman et al. *Osteoporos Int.* 2010 [38]	RET	– Upper extremity exercises: biceps curl, overhead triceps or press and upward row – Lower extremity exercises: back and knee extensions, side hip raise, and hip flexion and extension – Balance exercises: toe stand and heel stand	24 months	– 0–9 months: two home-based sessions/wk; – 10–24 months: two supervised sessions/wk	30–45 min. two sets x 8–12 repetitions of exercises	increased weights based on individual response, adding weights after two consecutive training sessions at the maximum set and repetition	– 0–9 months: home-based sessions – 10–24 months: supervised sessions	After chemotherapy and/or radiotherapy + Risedronate, calcium, and Vitamin D	Risedronate, calcium, and Vitamin D
Winters-Stone et al. *Breast Cancer Res Treat.* 2011 [39]	RET + IET	– IET: one to six jump sets (two-footed jumps from the ground to a target height 1 in. from the floor with a bent-knee landing, with weighted vests, x10 rep) – RET:one to two sets of three to four upper body, and three to four lower body exercises (with dumbbells for upper body, weighted vests for lower body, and a barbell for one combined upper/lower body exercise; wall-sits, 90° squats, bent-knee dead lifts, forward lunges, lateral lunges, 1-arm row, chest press, lateral raise, and push-ups). Home exercises were performed with the aid of elastic bands.	12 months	Two supervised session/wk + 1 home-based session/wk	45–60 min	RET: 60–70% of 1-RM for 1–3 sets of 8–12 rep; progressive increase by increasing band thickness, squat and lunge depth, and sets and repetitions	Supervised and home-based	After chemotherapy and/or radiotherapy	*Type of activity*: whole body stretching and relaxation exercises *Exercise modality*: in a seated or lying position to minimize weight-bearing forces *Protocol duration*: 12 months *Frequency*: 2 supervised session/wk + 1 home-based session/wk *Volume (session)*: 45–60 min *Intensity*: NR *Supervised and home-based* *After chemotherapy and/or radiotherapy*
Winters-Stone et al. *Osteoporos Int.* 2013 [40]	RET + IET	– IET: one to six jump sets (two-footed jumps from the ground to a target height 1 in. from the floor with a bent-knee landing, with weighted vests, x10 rep) – RET: one to two sets of three to four upper body, and three to four lower body exercises (with dumbbells for upper body, weighted vests for lower body, and a barbell for one combined upper/lower body exercise; wall-sits, 90° squats, bent-knee dead lifts, forward lunges, lateral lunges, 1-arm row, chest press, lateral raise, and push-ups). Home exercises were performed with the aid of elastic bands	12 months	Two supervised session/wk + 1 home-based session/wk	45–60 min	RET: 60–70% of 1-RM for 1–3 sets of 8–12 rep; progressive increase by increasing band thickness, squat and lunge depth, and sets and repetitions	Supervised and home-based	After chemotherapy and/or radiotherapy	*Type of activity*: whole body stretching and relaxation exercises *Exercise modality*: in a seated or lying position to minimize weight-bearing forces *Protocol duration*: 12 months *Frequency*: two supervised session/wk + one home-based session/wk *Volume (session)*: 45–60 min *Intensity*: NR *Supervised and home-based* *After chemotherapy and/or radiotherapy*

Abbreviations: 1RM: 1-repetition maximum; AET: aerobic exercise training; CG: control group; CET: combined exercise training; HR: heart rate; IG: intervention group; min: minutes; NR: not reported; RET: resistance exercise training; TCC: Tai Chi Chuan; WBV: whole body vibration; wk: week.

**Table 4:** Main results of the studies included.

Authors Journal Year	Results		
	*Intragroup Analysis—IG*	*Intragroup Analysis—CG*	*Intergroups Analysis*
Baker et al. *Integr. Cancer Ther.* 2018 [31]	*Molecular biomarkers* NTX/Cr (BCE/mmol Cr): 43.3 ± 20.0 vs. 45.9 ± 25.6; *p* = NR P1NP (μg/L): 62.3 ± 27.0 vs. 64.0 ± 25.6; *p* = NR	*Molecular biomarkers* NTX/Cr (BCE/mmol Cr): 39.0 ± 16.4 vs. 38.7 ± 12.9; *p* = NR P1NP (μg/L): 62.2 ± 25.3 vs. 59.5 ± 26.2; *p* = NR	*Molecular biomarkers* NTX/Cr (BCE/mmol Cr): 45.9 ± 25.6 vs. 38.7 ± 12.9; *p* = 0.929 P1NP (μg/L): 64.0 ± 25.6 vs. 59.5 ± 26.2; *p* = 0.286
De Paulo et al. *Exp. Gerontol.* 2018 [32]	*Molecular Biomarkers* CTX (ng/mL): 0.46 ± 0.2 vs. 0.46 ± 0.2; *p* = NS Osteocalcin (ng/mL): 19 ± 8 vs. 20 ± 9; *p*< 0.05 *Bone Mineral Density* Whole body BMD (g/cm^2^) 1.1 ± 0.1 vs. 1.1 ± 0.1; *p* = NS Lumbar spine BMD (g/cm^2^) 1.1 ± 0.1 vs. 1.0 ± 0.1; *p* = NS Total Hip BMD (g/cm^2^) 0.9 ± 0.1 vs. 0.9 ± 0.1; *p* = NS Trochanter BMD (g/cm^2^) 0.8 ± 0.1 vs. 0.9 ± 0.08; *p* = NS	*Molecular Biomarkers* CTX (ng/mL): 0.40 ± 0.1 vs. 0.33 ± 0.2; *p* = NS Osteocalcin (ng/mL): 17 ± 5 vs. 17 ± 6; *p* = NS *Bone Mineral Density* Whole body BMD (g/cm^2^) 1.1 ± 0.08 vs. 1.1 ± 0.1; *p* = NS Lumbar spine BMD (g/cm^2^) 1.1 ± 0.1 vs. 1.0 ± 0.1; *p* = NS Total Hip BMD (g/cm^2^) 0.9 ± 0.1 vs. 0.9 ± 0.1; *p* = NS Trochanter BMD (g/cm^2^) 0.9 ± 0.1 vs. 0.9 ± 0.1; *p* = NS	*Molecular Biomarkers* CTX (ng/mL): 0.46 ± 0.2 vs. 0.33 ± 0.2; *p* = 0.11 Osteocalcin (ng/mL): 20 ± 9 vs. 17 ± 6; *p* = 0.14 *Bone Mineral Density* Whole body BMD (g/cm^2^) 1.1 ± 0.1 vs. 1.1 ± 0.1; *p* = 0.5 Lumbar spine BMD (g/cm^2^) 1.0 ± 0.1 vs. 1.0 ± 0.1; *p* = 0.65 Total Hip BMD (g/cm^2^) 0.9 ± 0.1 vs. 0.9 ± 0.1; *p* = 0.54 Trochanter BMD (g/cm^2^) 0.9 ± 0.08 vs. 0.9 ± 0.1; *p* = 0.51
Dieli-Conwright et al. *Breast Cancer Res.* 2018 [33]	*Molecular Biomarkers* Osteocalcin (ng/mL): 12.1 ± 3.1 vs. 15.0 ± 4.1; *p* = 0.01 BSAP (ng/mL): 16.1 ± 4 vs. 18.0 ± 5.0; *p* = 0.01 CTX (ng/mL): 0.48 ± 0.1 vs. 0.44 ± 0.2; *p* = 0.07 NTX (nM BCE/L): 18.6 ± 3.1 vs. 17.7 ± 2.8; *p* = 0.10 RANK (pg/mL): 27.4 ± 6.8 vs. 26.7 ± 6.4; *p* = 0.14 RANKL (pmol/L): 142.5 ± 18.9 vs. 146.1 ± 16.1; *p* = 0.09 *Bone Mineral Density* Whole body BMD (g/cm^2^): 1.22 ± 0.1 vs. 1.27 ± 0.1; *p* = 0.15 Lumbar spine BMD (g/cm^2^): 1.16 ± 0.09 vs. 1.20 ± 0.09; *p* = 0.09 Total Hip BMD (g/cm^2^): 0.91 ± 0.09 vs. 0.94 ± 0.09; *p* = 0.17 Trochanter BMD (g/cm^2^): 0.72 ± 0.07 vs. 0.74 ± 0.07; *p* = 0.18 Femoral neck BMD (g/cm^2^): 0.88 ± 0.1 vs. 0.90 ±0.1; *p* = 0.21	*Molecular Biomarkers* Osteocalcin (ng/mL): 12.3 ± 3.4 vs. 12.0 ± 3.0; *p* = 0.61 BSAP (ng/mL): 16.2 ± 4.3 vs. 15.9 ± 4.2; *p* = 0.55 CTX (ng/mL): 0.47 ± 0.1 vs. 0.48 ± 0.2; *p* = 0.74 NTX (nM BCE/L): 18.4 ± 2.7 vs. 18.3 ± 2.5; *p* = 0.67 RANK (pg/mL): 26.9 ± 6.6 vs. 26.4 ± 6.5; *p* = 0.34 RANKL (pmol/L): 139.8 ± 18.1 vs. 148.8 ± 18.9; *p* = 0.47 *Bone Mineral Density* Whole body BMD (g/cm^2^): 1.20 ± 0.1 vs. 1.19 ± 0.1; *p* = 0.29 Lumbar spine BMD (g/cm^2^): 1.15 ± 0.09 vs. 1.14 ± 0.09; *p* = 0.57 Total Hip BMD (g/cm^2^): 0.90 ± 0.09 vs. 0.89 ± 0.08; *p* = 0.23 Trochanter BMD (g/cm^2^): 0.71 ± 0.06 vs. 0.70 ± 0.06; *p* = 0.43 Femoral neck BMD (g/cm^2^): 0.87 ± 0.1 vs. 0.86 ± 0.1; *p* = 0.23	*Molecular Biomarkers* Osteocalcin (ng/mL): 15.0 ± 4.1 vs. 12.0 ± 3.0; MD: 3.1 (5.6 to 1.5); *p* = 0.01 BSAP (ng/mL): 18.0 ± 5.0 vs. 15.9 ± 4.2; MD: 1.9 (2.4 to 0.55); *p* = 0.001 CTX (ng/mL): 0.44 ± 0.2 vs. 0.48 ± 0.2; MD: −0.04 (−0.10 to −0.06); *p* = 0.10 NTX (nM BCE/L): 17.7 ± 2.8 vs. 18.3 ± 2.5; MD: −0.90 (−1.1 to −0.6); *p* = 0.12 RANK (pg/mL): 26.7 ± 6.4 vs. 26.4 ± 6.5; MD: −0.70 (−0.9 to −0.4); *p* = 0.20 RANKL (pmol/L): 146.1 ± 16.1 vs. 148.8 ± 18.9; MD: 3.6 (5.1 to 1.2); *p* = 0.14 *Bone Mineral Density* Total BMD (g/cm^2^): 1.27 ± 0.1 vs. 1.19 ± 0.1; MD: 0.05 (0.04 to 0.02); *p* = 0.15 Lumbar spine BMD (g/cm^2^): 1.20 ± 0.09 vs. 1.14 ± 0.09; MD: 0.04 (0.03 to 0.01); *p* = 0.10 Hip BMD (g/cm^2^): 0.94 ± 0.09 vs. 0.89 ± 0.08; MD: 0.03 (0.03 to 0.00); *p* = 0.18 Trochanter BMD (g/cm^2^): 0.74 ± 0.07 vs. 0.70 ± 0.06; MD: 0.02 (0.03 to 0.00); *p* = 0.22 Femoral neck BMD (g/cm^2^): 0.90 ± 0.1 vs. 0.86 ± 0.1; MD: 0.02 (0.03 to 0.00); *p* = 0.21
Kim et al. *Cancer Nurs.* 2016 [34]	*Molecular biomarkers* NTX/Cr (nmol/mmol Cr): 45.98 ± 17.58 vs. 52.26 ± 17.78; *p* = NR *Bone mineral density* Lumbar spine BMD (g/cm^2^): 0.958 ± 0.080 vs. 0.966 ± 0.084; *p* = NR Total Hip BMD (g/cm^2^): 0.870 ± 0.089 vs. 0.876 ± 0.083; *p* = NR Femoral neck BMD (g/cm^2^): 0.797 ± 0.076 vs. 0.795 ± 0.075; *p* = NR	*Molecular biomarkers* NTX/Cr (nmol/mmol Cr): 47.85 ± 23.92 vs. 55.73 ± 26.86; *p* = NR *Bone mineral density* Lumbar spine BMD (g/cm^2^): 0.987 ± 0.064 vs. 0.985 ± 0.065; *p* = NR Total Hip BMD (g/cm^2^): 0.846 ± 0.075 vs. 0.845 ± 0.066; *p* = NR Femoral neck BMD (g/cm^2^): 0.798 ± 0.073 vs. 0.805 ± 0.080; *p* = NR	*Molecular biomarkers* NTX/Cr (nmol/mmol Cr): 52.26 ± 17.78 vs. 55.73 ± 26.86; *p* = 0.498 *Bone mineral density* Lumbar spine BMD (g/cm^2^): 0.966 ± 0.084 vs. 0.985 ± 0.065; *p* = 0.246 Total Hip BMD (g/cm^2^): 0.876 ± 0.083 vs. 0.845 ± 0.066; *p* = 0.506 Femoral neck BMD (g/cm^2^): 0.795 ± 0.075 vs. 0.805 ± 0.080; *p* = 0.352
Peppone et al. *Clin. Breast Cancer* 2010 [36]	*Molecular biomarkers* BSAP (μg/L): 8.34 ± 0.8 vs. 10.21 ± 1.1; *p* = NR NTX (nmBCE): 17.6 ± 3.7 vs. 11.1 ± 2.9; *p* = NR	*Molecular biomarkers* BSAP (μg/L): 7.64 ± 0.7 vs. 8.12 ± 1.1; *p* = NR NTX (nmBCE): 20.8 ± 3.3 vs. 18.8 ± 2.5; *p* = NR	*Molecular biomarkers* BSAP (μg/L): 10.21 ± 1.1 vs. 8.12 ± 1.1; *p* = 0.17 NTX (nmBCE): 11.1 ± 2.9 vs. 18.8 ± 2.5; *p* = 0.14
Peppone et al. *Support. Care Cancer* 2018 [35]	*Molecular biomarkers* NTX (nmBCE): 13 vs. 13.3; *p* = NR BSAP (ng/mL): 12.9 vs. 14.7; *p* = NR	*Molecular biomarkers* NTX (nmBCE): 12.8 vs. 14.3; *p* = NR BSAP (ng/mL): 12.2 vs. 13.8; *p* = NR	*Molecular biomarkers* NTX (nmBCE): 13.3 vs. 14.3; *p* = 0.86 BSAP (ng/mL): 14.7 vs. 13.8; *p* = 0.49
Tabatabai et al. *J. Clin. Endocrinol. Metab.* 2016 [37]	*Molecular biomarkers* Osteocalcin: NR (decreased); *p* < 0.05 P1NP: NR (decreased); *p* < 0.05 NTX/Cr: NR (decreased); *p* < 0.05 CTX: NR (decreased); *p* < 0.05 *Bone mineral density* Lumbar spine BMD (g/cm^2^): MD: 0.001 ± 0.005; *p* = NS Total Hip BMD (g/cm^2^): NR; NS Femoral BMD (g/cm^2^): NR; NS	*Molecular biomarkers* Osteocalcin: NR (decreased); *p* < 0.05 P1NP (decreased): NR; *p* < 0.05 NTX/Cr: NR (decreased); *p* < 0.05 CTX: NR (decreased); *p* < 0.05 *Bone mineral density* Lumbar spine BMD (g/cm^2^): MD: −0.014 ± 0.005; *p* = 0.03 Total Hip BMD (g/cm^2^): NR; NS Femoral BMD (g/cm^2^): NR; NS	*Molecular biomarkers* Osteocalcin: NR; NS P1NP: NR; NS NTX/Cr: NR; NS CTX: NR; NS *Bone mineral density* Femoral BMD (g/cm^2^): NR; NS Lumbar spine BMD (g/cm^2^): NR; NS Hip BMD (g/cm^2^): NR; NS
Waltman et al. *Osteoporos Int.* 2010 [38]	*Molecular biomarkers* BSAP (%MD): −11.10% ± 2.3; *p* < 0.001 NTX (%MD): −23.20% ± 2.8; *p* < 0.001 *Bone mineral density* Lumbar spine BMD (%MD): 3.08 ± 0.44; *p* < 0.0001 Total Hip BMD (%MD): 2.15 ± 0.28; *p* < 0.0001 Femoral neck BMD (%MD): 0.92 ± 0.50; *p* = 0.06 Radius (33%) BMD (%MD): −0.18 ± 0.41; *p* = 0.66 Total radius BMD (%MD): −0.27 ± 0.60; *p* = 0.66	*Molecular biomarkers* BSAP (%MD): −08.70 ± 2.6; *p* < 0.001 NTX (%MD): −16.70 ± 3.3; *p* < 0.001 *Bone mineral density* Lumbar spine BMD (%MD): 2.85 ± 0.40; *p* < 0.0001 Total Hip BMD (%MD): 1.81 ± 0.36; *p* < 0.0001 Femoral neck BMD (%MD): 0.63 ± 0.42; *p* = 0.14 Radius (33%) BMD (%MD): −0.16 ± 0.56; *p* = 0.77 Total radius BMD (%MD): −0.57 ± 0.61; *p* = 0.35	*Molecular biomarkers* BSAP (%MD): −11.10 ± 2.3 vs. −08.70 ± 2.6; *p* = NR NTX (%MD):−23.20 ± 2.8 vs. −16.70 ± 3.3; *p* = NR *Bone mineral density* Lumbar spine BMD (%MD): 3.08 ± 0.44 vs. 2.85 ± 0.40; *p* = NR Total Hip BMD (%MD): 2.15 ± 0.28 vs. 1.81 ± 0.36; *p* = NR Femoral neck BMD (%MD):0.92 ± 0.50 vs. 0.63 ± 0.42; *p* = NR Radius (33%) BMD (%MD): −0.18 ± 0.41 vs. −0.16 ± 0.56; *p* = NR Total radius BMD (%MD): −0.27 ± 0.60 vs. −0.57 ±0.61; *p* = NR
Winters-Stone et al. *Breast Cancer Res Treat.* 2011 [39]	*Molecular biomarkers* Osteocalcin (ng/mL): 12.6 ± 4.4 vs. 12.8 ± 3.8; *p* = NR Deoxypyridinoline (mMol/mMolCr): 21.4 ± 9.8 vs. 13.1 ± 4.2; *p* = NR *Bone mineral density* Lumbar spine BMD (g/cm^2^): 0.983 ± 0.146 vs. 0.987 ± 0.146; *p* = NR Total Hip BMD (g/cm^2^): 0.863 ± 0.101 vs. 0.860 ± 0.105; *p* = NR Trochanter BMD (g/cm^2^): 0.657 ± 0.088 vs. 0.654 ± 0.087; *p* = NR Femoral neck BMD (g/cm^2^): 0.731 ± 0.100 vs. 0.721 ± 0.101; *p* = NR	*Molecular biomarkers* Osteocalcin (ng/mL): 11.3 ± 4.1 vs. 14.3 ± 5.0; *p* = NR Deoxypyridinoline (mMol/mMolCr): 17.1 ± 5.6 vs. 12.2 ± 3.1; *p* = NR *Bone mineral density* Lumbar spine BMD (g/cm^2^): 0.971 ± 0.120 vs. 0.949 ± 0.108; *p* = NR Total Hip BMD (g/cm^2^): 0.848 ± 0.099 vs. 0.841 ± 0.096; *p* = NR Trochanter BMD (g/cm^2^): 0.642 ± 0.091 vs. 0.641 ± 0.089; *p* = NR Femoral neck BMD (g/cm^2^): 0.728 ± 0.091 vs. 0.713 ± 0.082; *p* = NR	*Molecular biomarkers* Osteocalcin (ng/mL): 12.8 ± 3.8 vs. 14.3 ± 5.0; *p* = 0.01 Deoxypyridinoline (mMol/mMolCr): 13.1 ± 4.2 vs. 12.2 ± 3.1; *p* = 0.22 *Bone mineral density* Lumbar spine BMD (g/cm^2^): 0.987 ± 0.146 vs. 0.949 ± 0.108; *p* < 0.01 Total Hip BMD (g/cm^2^): 0.860 ± 0.105 vs. 0.841 ± 0.096; *p* = 0.13 Trochanter BMD (g/cm^2^): 0.654 ± 0.087 vs. 0.641 ± 0.089; *p* = 0.15 Femoral neck BMD (g/cm^2^): 0.721 ± 0.101 vs. 0.713 ± 0.082; *p* = 0.27
Winters-Stone et al. *Osteoporos Int.* 2013 [40]	*Molecular biomarkers* Osteocalcin (ng/mL): 10.6 ± 4.07 vs. 9.78 ± 4.5; *p* = NR Deoxypyrodinoline (nmol/mmol Cr): 13.0 ± 4.95 vs. 15.8 ± 12.1; *p* = NR *Bone mineral density* Lumbar spine BMD (g/cm^2^): 0.983 ± 0.113 vs. 0.972 ± 0.119; *p* = NR Total Hip BMD (g/cm^2^): 0.909 ± 0.095 vs. 0.899 ± 0.096; *p* = NR Trochanter BMD (g/cm^2^): 0.689 ± 0.065 vs. 0.683 ± 0.066; *p* = NR Femoral neck BMD (g/cm^2^): 0.809 ± 0.11 vs. 0.804 ± 0.108; *p* = NR	*Molecular biomarkers* Osteocalcin (ng/mL): 14.0 ± 3.78 vs. 11.9 ± 5.5; *p* = NR Deoxypyrodinoline (nmol/mmol Cr): 14.2 ± 4.57 vs. 15.0 ± 8.6; *p* = NR *Bone mineral density* Lumbar spine BMD (g/cm^2^): 0.988 ± 0.118 vs. 0.970 ± 0.126; *p* = NR Total Hip BMD (g/cm^2^): 0.892 ± 0.119 vs. 0.887 ± 0.119; *p* = NR Trochanter BMD (g/cm^2^): 0.666 ± 0.099 vs. 0.662 ± 0.101; *p* = NR Femoral neck BMD (g/cm^2^): 0.781 ± 0.093 vs. 0.773 ± 0.095; *p* = NR	*Molecular biomarkers* Osteocalcin (ng/mL): 9.78 ± 4.5 vs. 11.9 ± 5.5; *p* = 0.22 Deoxypyrodinoline (nmol/mmol Cr): 15.8 ± 12.1 vs. 15.0 ± 8.6; *p* = 0.39 *Bone mineral density* Lumbar spine BMD (g/cm^2^): 0.972 ± 0.119 vs. 0.970 ± 0.126 *p* = 0.18 Total Hip BMD (g/cm^2^): 0.899 ± 0.096 vs. 0.887 ± 0.887; *p* = 0.65 Trochanter BMD (g/cm^2^): 0.683 ± 0.066 vs. 0.662 ± 0.101; *p* = 0.90 Femoral neck BMD (g/cm^2^): 0.804 ± 0.108 vs. 0.773 ± 0.095; *p* = 0.68

Abbreviations: Alkphase B: bone-specific alkaline phosphatase; BCE/mMol Cr: bone collagen equivalent per mMol creatinine; BMD: bone mineral density; BSAP: bone specific alkaline phosphatase; CG: control group; CTX: C-telopeptides of type I collagen; IG: intervention group; MD: mean difference; NR: not reported; NS: not significant; NTX/Cr: N-telopeptide X/creatinine; NTX: crosslinked N-telopeptides of type I collagen; P1NP: serum type 1 procollagen N-terminal propeptide; RANK(L): receptor activator of nuclear factor (ligand); SERM: selective estrogen receptor modulator.

**Table 5:** Quality assessment of the studies included in the present systematic review.

*Articles*	*Domain*					*Score*
	*Random Sequence Generation*	*Appropriate* *Randomization*	*Blinding of Participants or Personnel*	*Blinding of Outcome Assessors*	*Withdrawals and Dropouts*	
Baker et al., 2018 [31]	1	1	0	1	1	4
De Paulo et al., 2018 [32]	1	0	0	0	1	2
Dieli-Conwright et al., 2018 [33]	1	1	0	0	1	3
Kim et al., 2016 [34]	1	1	0	1	1	4
Peppone et al., 2010 [35]	1	1	0	0	1	3
Peppone et al., 2018 [36]	1	1	0	0	1	3
Tabatabai et al., 2016 [37]	1	1	0	1	1	4
Waltman et al., 2010 [38]	1	0	0	0	1	2
Winters-Stone et al., 2011 [39]	1	0	0	1	1	3
Winters-Stone et al., 2013 [40]	1	0	0	1	1	3

Points were awarded as follows: study described as randomized, 1 point; appropriate randomization, 1 point; subjects blinded to intervention, 1 point; evaluator blinded to intervention, 1 point; description of withdrawals and dropouts, 1 point.

## Data Availability

The data presented in this study are available on request from the corresponding author.

## Supplementary Materials

The following supporting information can be downloaded at: https://www.mdpi.com/article/10.3390/ijms24020921/s1.
