# Supplementary material for: Impact of Physical Rehabilitation on Bone Biomarkers in Non-Metastatic Breast Cancer Women: A Systematic Review and Meta-Analysis
Source: Int J Mol Sci. 2023 Jan 4;24(2):921. doi: 10.3390/ijms24020921 (PMC9863706; doi:10.3390/ijms24020921)
Supplement: Supplementary file 1 [file ijms-24-00921-s001.zip › ijms-2074077-supplementary.pdf]

Supplementary Table S1. Characteristics of excluded studies assessed in full-text.

Study	*Reason for exclusion*
Bloom et al., 2013	Abstract
Dieli-Conwright et al., 2018	Not relevant bone biomarkers
Dobek et al.,	Not RCT
Foulkers et al., 2020	Study protocol
Knobf et al., 2016	No homogeneous sample of BC patients
Liu et al., 2022	Not RCT
Love et al., 1994	No rehabilitation intervention
NCT03712813	Study protocol
Nikander et al., 2007	Not relevant bone biomarkers
Nikander et al., 2012	Not relevant bone biomarkers
Rogers et al., 2009	Not relevant bone biomarkers
Saarto et al., 2011	Not relevant bone biomarkers
Swenson et al., 2009	Abstract
Tabatabai et al., 2016	Not RCT
Thomas et al., 2016	Not relevant bone biomarkers
Toriola et al., 2015	Not RCT
Uth et al., 2021	Not relevant bone biomarkers
Vehmanen et al., 2021	Not relevant bone biomarkers
Winters-stone et al., 2012	Not RCT
